# Omitting re-excision for focally positive margins after breast-conserving surgery does not impair disease-free and overall survival

**DOI:** 10.1007/s10549-017-4232-6

**Published:** 2017-04-07

**Authors:** Elvira L. Vos, Sabine Siesling, Margreet H.A. Baaijens, Cornelis Verhoef, Agnes Jager, Adri C. Voogd, Linetta B. Koppert

**Affiliations:** 1 Department of Oncological Surgery, Erasmus MC Cancer Institute, DHA-102, PO Box 2400, 3000 Rotterdam, The Netherlands; 2 Department of Research, Netherlands Comprehensive Cancer Organisation (IKNL), PO Box 19079, 3501 Utrecht, The Netherlands; 3 Department of Health Technology and Services Research, MIRA Institute for Biomedical Technology and Technical Medicine, University of Twente, PO Box 217, 7500 Enschede, The Netherlands; 4 Department of Radiotherapy, Erasmus MC Cancer Institute, PO Box 5201, 3008 Rotterdam, The Netherlands; 5 Department of Medical Oncology, Erasmus MC Cancer Institute, PO Box 5201, 3008 Rotterdam, The Netherlands; 6 Department of Epidemiology, Maastricht University, PO Box 616, 6200 Maastricht, The Netherlands

**Keywords:** Breast-conserving surgery, Resection margins, Re-excision, Re-operation, Ipsilateral breast tumor recurrence, Disease-free survival, Overall survival

## Abstract

**Purpose:**

In contrast to other countries, the Dutch breast cancer guideline does not recommend re-excision for focally positive margins after breast-conserving surgery (BCS) in invasive tumor and does recommend whole-breast irradiation including boost. We investigated whether omitting re-excision as compared to performing re-excision affects prognosis with a retrospective population-based cohort study.

**Methods:**

The total cohort included 32,119 women with primary BCS for T1–T3 breast cancer diagnosed between 2003 and 2008 from the nationwide Netherlands cancer registry. The subcohort included 10,433 patients in whom the resection margins were registered. Outcome measures were 5-year ipsilateral breast tumor recurrence (IBTR) rate, 5-year disease-free survival (DFS) rate, and 10-year overall survival (OS) rate.

**Results:**

In the total cohort, 25,878 (80.6%) did not have re-excision, 2368 (7.4%) had re-excision by BCS, and 3873 (12.1%) had re-excision by mastectomy. Five-year IBTR rates were 2.1, 2.8, and 2.9%, respectively (*p* = 0.001). In the subcohort, 7820 (75.0%) had negative margins without re-excision, 492 (4.7%) had focally positive margins without re-excision, 586 (5.6%) had focally positive margins and underwent re-excision, and 1535 (14.7%) had extensively positive margins and underwent re-excision. Five-year IBTR rate was 2.3, 2.9, 1.1, and 2.9%, respectively (*p* = 0.099). Compared to omitting re-excision, performing re-excision for focally positive margins was associated with lower risk of IBTR (adjusted HR 0.30, 95% CI 0.11–0.82), but not with DFS (adjusted HR 0.83 95% CI 0.59–1.17) nor with OS (adjusted HR 1.17 95% CI 0.87–1.59).

**Conclusion:**

Omitting re-excision in breast cancer patients for focally positive margins after BCS does not impair DFS and OS, provided that whole-breast irradiation including boost is given.

**Electronic supplementary material:**

The online version of this article (doi:10.1007/s10549-017-4232-6) contains supplementary material, which is available to authorized users.

## Introduction

For breast-conserving surgery (BCS), the minimally accepted resection margin above which a re-excision will be advised has been debated extensively since high level of evidence is lacking. For many years, the international debate has been predominated by finding the ideal negative margin width, whereby it was compared to positive margins in general. A meta-analysis concluded that there is no evidence that increasing the tumor-free margin width significantly reduces the odds of local recurrence [1]. Since a tumor-positive margin did increase the odds of local recurrence, the Society of Surgical Oncology and the American Society for Radiation Oncology (SSO-ASTRO) and European Society for Medical Oncology (ESMO) recently published guidelines recommending *no ink on tumor* as an adequate margin for invasive breast cancer. Re-excision is only advised in case the tumor is touching the inked resection margin [2, 3]. No distinction was made, however, between focally and extensively positive margins.

The next question in the debate is whether a focally positive margin is a risk factor for local recurrence and therefore an indication for re-excision. Six out of seven studies previously investigating local recurrence rate in patients with focally positive margins after final surgery found it was not different from negative margins [4–10]. In the last decade, the risk of local recurrence has decreased even more through improvements in radiotherapy and systemic treatment [11]. The impact of margin status on local relapse was also investigated in the European Organisation for Research and Treatment of Cancer (EORTC) boost versus no boost trial. Margin status had no significant influence, suggesting that radiotherapy boost on the tumor bed negates the prognostic significance of positive margins [12, 13].

Uniquely, since 2002, the Dutch national guideline does not recommend a re-excision for focally positive margins after BCS in case of invasive disease and does recommends to apply whole-breast radiotherapy including a boost on the tumor bed in this situation [14]. As far as we know, the Netherlands is the only country with such a liberal approach. How often re-excision is indeed omitted in clinical practice is unknown [15]. The aim of the current study was to describe the implementation of the recommendation in clinical practice and investigate whether omitting re-excision for focally positive margins affects ipsilateral breast tumor recurrence (IBTR), disease-free survival (DFS), and overall survival (OS) in a nationwide cancer registry.

## Methods

**Fig. 1:**
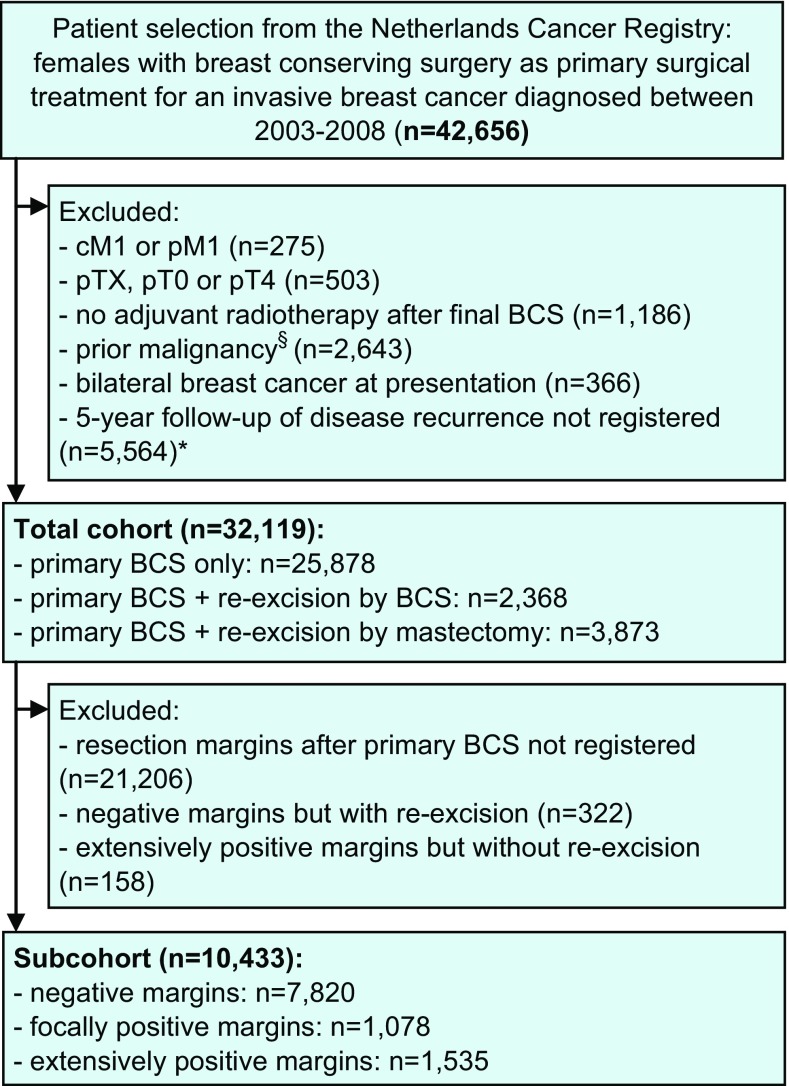
Patient selection. *cM1* clinically suspect distant metastasis, *pM1* pathologically confirmed distant metastasis, *pTX* primary tumor cannot be assessed or is unknown, *pT0* no evidence of primary tumor, *pT4* tumor with direct extension to the chest wall and/or to the skin, *BCS* breast-conserving surgery. ^§^Except for basal-like skin cancer. ^*^Follow-up information on disease recurrence was assembled for all patients diagnosed in 2003–2006, and for 44% of the patients diagnosed in 2007–2008 due to lack of funding for some hospitals. ^†^All patients of whom the breast was conserved had adjuvant radiotherapy. From all patients with a re-excision by mastectomy, 873 (22.5%) had adjuvant radiotherapy. ^‡^Since it was an optional item in the coding manual, some registration clerks did not code margins

In this retrospective population-based cohort study, all female invasive breast cancer patients diagnosed between 2003 and 2008 with BCS as their primary surgical treatment in the Netherlands were included. Data were retrieved from the Netherlands Cancer Registry that includes all new cancer diagnoses in the Netherlands since 1989 covering 17 million inhabitants. The main source of information is the national pathology archive and in addition the registry is linked with the national discharge register. Specially trained registration clerks from the Netherlands Cancer Registry are located in each hospital in the Netherlands, both academic and non-academic, and independently collect data on patient demographics, tumor characteristics, and breast cancer treatment. Data completeness exceeds 95% [16]. The registration clerks follow a strict coding manual of which the majority is mandatory to register. Registration of resection margins was optional and it was not collected by all registration clerks. Death certificates are not available, due to privacy regulations. Vital status and date of death are obtained by a yearly linkage to the Municipal Personal Records Database and are complete up to December 31, 2014. Information on local, regional, and contralateral recurrence as well as distant metastasis is not collected routinely by the cancer registry. On a project basis, it was collected retrospectively by the local registration clerks up to 5 years after primary breast cancer diagnosis for the cohort diagnosed between 2003 and 2008. The exclusion criteria are shown in Fig. 1.

## Definitions

Clinical and pathological tumor node metastasis staging (TNM) was in accordance with the sixth edition of TNM Classification of Malignant Tumors by the American Joint Committee on Cancer (AJCC). Surgical treatment was classified as ‘primary BCS only’ for patients who underwent BCS not followed by a re-excision. Patients who underwent BCS followed by a re-excision were classified as ‘re-excision by BCS’ or ‘re-excision by mastectomy’ according to the type of final re-excision. Margin status was classified as ‘negative’ defined as no invasive tumor component and/or adjacent DCIS component touching the inked margin, ‘focally positive’ defined as foci of invasive tumor component and/or adjacent DCIS component touching the inked margin over a length of four mm or less, or ‘extensively positive’ defined as foci of invasive tumor component and/or adjacent DCIS component touching the inked margin over a length of more than four mm. The four-mm cut-off is a translation from the previous definition of three low-power microscopic fields (using a ×10 ocular lens). All Dutch pathologists are obliged to report the margin status by this classification. The use of radiotherapy was registered as yes or no, but details about the total dose, anatomical fields, and use of boost were not included in the registry.

The first recurrence that occurred at least 3 months from primary breast cancer diagnosis was registered by the Netherlands Cancer Registry. Other recurrence(s) within 3 months from the first recurrence were also included. IBTR rate was defined as the percentage of patients with ipsilateral local recurrence of breast carcinoma. DFS rate was defined as the percentage of patients being alive without having had any breast cancer recurrence (i.e., local, regional, contralateral, or distant). OS rate was defined as the percentage of patients being alive.

### Dutch national breast cancer guideline

The Dutch guideline is evidence-based and complemented with expert opinion written by a multidisciplinary team and is regularly updated. The goal is to advise and guide clinical practice. In the timeframe studied, it recommended whole-breast irradiation with a doses equivalence of 50 Gy followed by a 14–16 Gy boost in case of negative margins. Boost could be omitted in patients older than 60 years. In case of focally positive margins or patients being 40 years or younger with negative margins, a 20–25 Gy boost was recommended. Post-mastectomy chest wall irradiation was recommended in case of positive margins, tumor growth into the pectoral muscle, and should be considered for T3 tumors, with a doses equivalence of 45–50 or 60–70 Gy in case of macroscopic residual tumor. Regional lymph node irradiation was indicated in case of pN2 or if the highest axillary medial node was positive.

### Statistical analysis

To avoid noise in the comparisons between groups that are defined by the patient’s margin status and surgical treatment, patients were excluded if they had re-excision for negative margins and if they did not had re-excision for extensively positive margins (Fig. 1). Primary outcome was IBTR and secondary outcomes were DFS and OS. The effect of re-excision and type of re-excision (i.e., BCS or mastectomy) on the outcomes was studied in the total cohort (32,119 patients), irrespective of the resection margins after primary BCS. Subsequently, the effect of resection margins on the outcomes was studied in a subcohort (10,433 patients) of whom the resection margins after primary BCS were registered (Fig. 1). Time of follow-up was defined as the time between the latest re-excision and the event or censoring. Patients were censored in case of emigration, 5 years after the latest breast cancer operation concerning IBTR and DFS, or at the December 31, 2014 concerning OS. Differences in patient characteristics were tested using the *χ* ^2^ test. IBTR rate, DFS rate, and OS rate were determined by Kaplan–Meier method and distributions between subgroups were compared by the log-rank test. Hazard ratios (HR) were estimated by Cox proportional hazards regression analysis. In the total cohort, comparisons were made with respect to whether or not a re-excision was performed. In the subcohort, comparisons were made with respect to resection margins including whether or not a re-excision was performed. Due to the prognostic importance of systemic therapy, the effect of margin status on IBTR was also studied after stratification for use of (neo)adjuvant systemic therapy (none versus endocrine therapy and/or chemotherapy). Multivariable models were performed by the enter method (i.e., including all covariates at the same time in the model and no forward or backward selection) and included all clinicopathological and treatment variables with a maximum degrees of freedom of ten events per covariate included. Missing values were classified as unknown. In spite of missing values, all patients were included in the analyses to prevent bias in IBTR, DFS, and OS rate estimates. Interaction was tested between all variables and margin status for IBTR. The proportional hazards assumption was tested by graphing the log(−log(IBTR)) versus log of IBTR time of each variable in Table 1 and was considered proportional when parallel curves were observed. Statistical tests were two-sided, and *p* value <0.050 was considered statistically significant. SPSS^®^ version 21 (IBM, Armonk, New York, USA) was used for all statistical analyses.

**Table 1:** Clinicopathological and treatment characteristics according to the performance of re-excision in the total cohort (*n* = 32119)

	Primary BCS only	Primary BCS + re-excision by BCS	Primary BCS + re-excision by mastectomy	*p* value^a^
No. of patients	25878 (80.6)	2368 (7.4)	3873 (12.1)	
Age				<0.001
>60 years	10505 (40.6)	718 (30.3)	1185 (30.6)	
51–60 years	7946 (30.7)	780 (32.9)	1113 (28.7)	
41–50 years	5795 (22.4)	681 (28.8)	1152 (29.7)	
≤40 years	1632 (6.3)	189 (8.0)	423 (10.9)	
Histology				<0.001
Ductal	20148 (77.9)	1797 (77.9)	2548 (65.8)	
Lobular^b^	2841 (11.0)	325 (13.7)	901 (23.3)	
Other	2889 (11.2)	246 (10.4)	424 (10.9)	
Differentiation grade				<0.001
1	6657 (25.7)	550 (23.2)	606 (15.6)	
2	10726 (41.4)	985 (41.6)	1611 (41.6)	
3	7086 (27.4)	657 (27.7)	1281 (33.1)	
Unknown	1409 (5.4)	176 (7.4)	375 (9.7)	
pT				<0.001
T1	19289 (74.5)	1730 (73.1)	2164 (55.9)	
T2	6463 (25.0)	631 (26.6)	1493 (38.5)	
T3	51 (0.2)	6 (0.3)	216 (5.6)	
ypT0	75 (0.3)	1 (0.0)	–	
pN				<0.001
N0	18200 (70.3)	1589 (67.1)	1998 (51.6)	
N1	5944 (23.0)	620 (26.2)	1284 (33.2)	
N2	1045 (4.0)	104 (4.4)	375 (9.7)	
N3	432 (1.7)	35 (1.5)	183 (4.7)	
Unknown	257 (1.0)	20 (0.8)	33 (0.9)	
Oestrogen receptor				<0.001
Positive	13508 (52.2)	1267 (53.5)	1803 (46.6)	
Negative	2720 (10.5)	230 (9.7)	422 (10.9)	
Unknown	9650 (37.3)	871 (36.8)	1648 (42.6)	
Progesterone receptor				<0.001
Positive	10836 (41.9)	996 (42.1)	1414 (36.5)	
Negative	4874 (18.8)	424 (17.9)	725 (18.7)	
Unknown	10168 (39.3)	948 (40.0)	1734 (44.8)	
HER2Neu receptor				<0.001
Negative	13282 (51.3)	1220 (51.5)	1647 (42.5)	
Positive	1761 (6.8)	183 (7.7)	404 (10.4)	
Unknown	10835 (41.9)	965 (40.8)	1822 (47.0)	
Systemic therapy^c^				<0.001
None	13128 (51.1)	1114 (47.0)	1321 (34.1)	
Chemotherapy only	3173 (12.3)	286 (12.1)	565 (14.6)	
Hormonal therapy only	4038 (15.6)	332 (14.0)	616 (15.9)	
Chemotherapy and hormonal therapy	5449 (21.1)	636 (26.9)	1371 (35.4)	

*pT* pathological tumor stage, *pN* regional lymph nodes stage

^a^ *χ* ^2^ test

^b^Includes mixed ductal and lobular tumors

^c^Both neoadjuvant and adjuvant

## Results

### Re-excision in total cohort

Of the total of 42,656 women with invasive breast cancer diagnosed between 2003 and 2008 and primarily treated with BCS in the Netherlands, 32,119 met the eligibility criteria. Patient selection is displayed in Fig. 1. Patients in whom no 5-year follow-up was collected due to lack of funding did not differ from the total cohort in terms of clinicopathological and treatment characteristics. Re-excision was performed in 6241 (19.4%) patients of whom 3873 (62.1%) underwent a mastectomy. The frequency of mastectomy as the re-excision decreased over time from 65.9% in 2003 to 52.5% in 2008. Clinicopathological and treatment characteristics are shown in Table 1. All patients with primary BCS only and re-excision by BCS have had radiotherapy. From the 3873 patients with re-excision by mastectomy, 873 (22.5%) had radiotherapy. Median follow-up time for IBTR, DFS, and OS was 60 months (interquartile range (IQR) 57–60), 60 months (IQR 57–60), and 106 months (86–123), respectively. After testing the proportional hazards assumption for all variables as shown in Table 1, a constant relative hazard was seen for IBTR risk and therefore time was not included in the multivariable models. The 5-year IBTR rate in the primary BCS only, re-excision by BCS, and re-excision by mastectomy group was 2.1, 2.8, and 2.9%, respectively (*p* = 0.001) (Table 2). Multivariable analysis showed that IBTR rates after re-excision by BCS and re-excision by mastectomy were not statistically significantly different as compared to primary BCS only (HR 1.31 95% CI 1.00–1.71 and HR 0.89 95% CI 0.57–1.40, respectively). DFS rates and OS rates are shown in Table 2 and were statistically significantly decreased in patients with re-excision by mastectomy as compared to primary BCS only after multivariable analyses.

**Table 2:** Ipsilateral breast tumor recurrence (IBTR), disease-free survival (DFS), and overall survival (OS) rates from Kaplan–Meier analysis and adjusted hazard ratio (HR) from multivariable Cox regression analysis according to the performance of re-excision in the total cohort (*n* = 32119)

	IBTR		DFS		OS		
	5-year (%)	Adjusted^a^ HR (95% CI)	5-year (%)	Adjusted^a^ HR (95% CI)	5-year (%)	10-year (%)	Adjusted^a^ HR (95% CI)
Primary BCS only (*n* = 25878)^b^	2.1	Reference	89.0	Reference	92.7	82.1	Reference
Primary BCS + re**-**excision by BCS (*n* = 2368)^b^	2.8	1.31 (1.00–1.71)	87.7	1.13 (1.00–1.28)	94.5	85.3	0.89 (0.80–1.00)
Primary BCS + re-excision by mastectomy (*n* = 3873)^c^	2.9	0.89 (0.57–1.40)	82.9	*1*.*38 (1*.*19*–*1*.*61)*	89.9	78.7	*1*.*16 (1*.*01*–*1*.*34)*

Italic values indicate* p* < 0.05

^a^Adjusted for: age (continuous), histology (ductal, lobular, or other), differentiation grade (1, 2, 3, or unknown), pT stage (1, 2, 3, or ypT0), pN stage (1, 2, 3, or unknown), estrogen receptor status (positive, negative, or unknown), HER2Neu receptor status (positive, negative, or unknown), use of systemic therapy (any or none), and radiotherapy (yes or no)

^b^All patients had radiotherapy (100%)

^c^Radiotherapy was performed in 873 (22.5%) of the patients

### Resection margins in subcohort

**Fig. 2:**
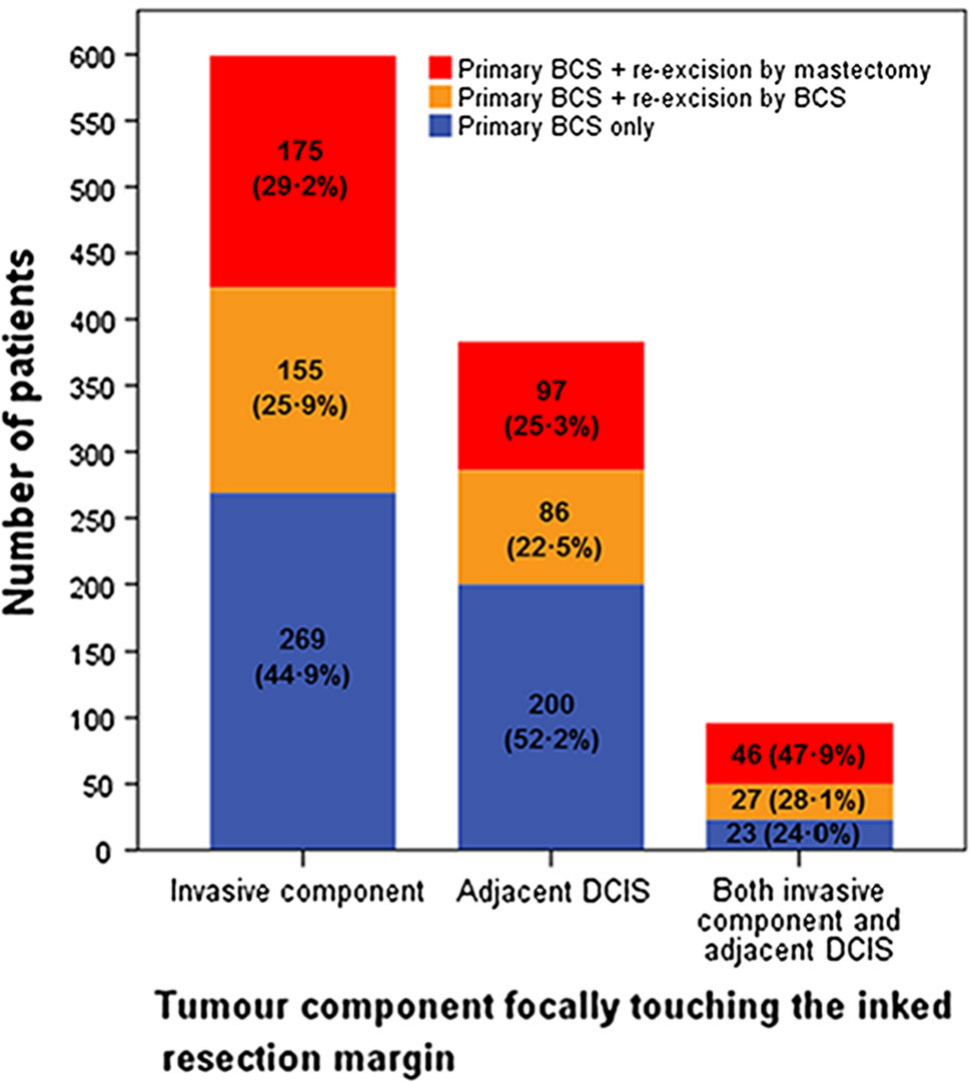
Re-excisions according to the tumor component focally touching the inked margin. Figure only shows the patients with focally positive margins after primary BCS from the subcohort (*n* = 1078). Re-excision was omitted in 492 (45.6%) patients (total of *blue bars*) and was performed in 586 (54.4%) patients (total of *orange* and *red bars*). The frequency and type of re-excision is shown according to which component of the tumor was focally touching the inked margin

The subcohort consisted of 10,433 (32.5%) patients (Fig. 1). The registration of resection margin in the cancer registry was not associated with the occurrence of IBTR (OR 1.12 95% CI 0.95–1.31 *p* = 0.180). Clinicopathological and treatment characteristics are shown in Table 3. Negative margins were observed in 7820 (75.0%) patients, focally positive margins in 1078 (10.3%) patients, and extensively positive margins in 1535 (14.7%) patients. After focally positive margins, in 492 (45.6%) patients, re-excision was omitted (i.e., primary BCS only) and in 586 (54.4%) patients re-excision was performed. The frequency of omitting the re-excision varied non-linearly over time ranging between 32.8 and 58.4% and the proportional hazards assumption for IBTR was not violated. Figure 2 shows the use of re-excision in the patients with focally positive margins according to whether the invasive component and/or the adjacent DCIS component were focally touching the inked margins. Of the 586 patients with focally positive margins and re-excision, 268 (45.7%) underwent a re-excision by BCS followed by adjuvant radiotherapy and 318 (54.3%) underwent a re-excision by mastectomy of whom 84 (26.4%) had post-mastectomy radiotherapy. The type of re-excision varied non-linearly over time with the frequency of BCS ranging between 40.7 and 53.3%.

**Table 3:** Clinicopathological and treatment characteristics according to resection margin after primary BCS and the performance of re-excision in the subcohort (*n* = 10433)

	Negative margin and primary BCS only	Focally positive margin and primary BCS only	Focally positive margin and primary BCS + re-excision^a^	Extensively positive margin and primary BCS + re-excision^b^	*p* value
Total no. of patients	7820 (75.0)	492 (4.7)	586 (5.6)	1535 (14.7)	
Age					<0.001
>60 years	2695 (34.5)	210 (42.7)	149 (25.4)	374 (24.4)	
51–60 years	2346 (30.0)	120 (24.4)	169 (28.8)	417 (27.2)	
41–50 years	2258 (28.9)	145 (29.5)	218 (37.2)	583 (38.0)	
≤40 years	521 (6.7)	17 (3.5)	50 (8.4)	161 (10.5)	
Histology					<0.001
Ductal	6233 (79.7)	379 (77.0)	427 (72.9)	1046 (68.2)	
Lobular^c^	783 (10.0)	69 (13.8)	110 (18.8)	317 (20.6)	
Other	804 (10.3)	45 (9.1)	49 (8.4)	172 (11.2)	
Differentiation grade					<0.001
1	1766 (22.6)	105 (21.3)	94 (16.0)	254 (16.5)	
2	3114 (39.8)	202 (41.1)	229 (39.1)	618 (40.3)	
3	2440 (31.2)	149 (30.3)	217 (37.0)	506 (33.0)	
Unknown	500 (6.4)	36 (7.3)	46 (7.8)	157 (10.2)	
pT					<0.001
T1	5576 (71.3)	345 (70.1)	347 (59.2)	866 (56.4)	
T2	2123 (27.1)	145 (29.5)	227 (38.7)	593 (38.6)	
T3	15 (0.2)	2 (0.4)	12 (2.0)	76 (5.0)	
ypT0	34 (0.4)	–	–	–	
pN					<0.001
N0	5166 (66.1)	316 (64.2)	297 (50.7)	816 (53.2)	
N1	2231 (27.4)	117 (23.8)	214 (36.5)	503 (32.8)	
N2	344 (4.4)	40 (8.1)	47 (8.0)	142 (9.3)	
N3	120 (1.5)	17 (3.5)	25 (4.3)	61 (3.9)	
Unknown	67 (0.9)	2 (0.4)	3 (0.5)	13 (0.8)	
Oestrogen receptor					<0.001
Positive	3292 (42.1)	217 (44.1)	245 (41.8)	531 (34.6)	
Negative	710 (9.1)	42 (8.5)	55 (9.4)	124 (8.1)	
Unknown	3818 (48.8)	233 (47.4)	286 (48.8)	880 (57.3)	
Progesterone receptor					<0.001
Positive	2662 (34.0)	176 (35.8)	182 (31.1)	410 (26.7)	
Negative	1253 (16.0)	81 (16.5)	103 (17.6)	227 (14.8)	
Unknown	3905 (49.9)	235 (47.8)	301 (51.4)	898 (58.5)	
HER2Neu receptor					<0.001
Negative	3890 (37.0)	188 (38.2)	196 (33.4)	392 (25.5)	
Positive	980 (12.5)	68 (13.8)	96 (16.4)	233 (15.2)	
Unknown	3950 (50.5)	236 (48.0)	294 (50.2)	910 (59.3)	
Systemic therapy^d^					<0.001
None	3214 (41.1)	183 (37.2)	179 (29.0)	468 (30.5)	
Chemotherapy only	1011 (12.9)	53 (10.8)	82 (14.0)	225 (14.7)	
Hormonal therapy only	1168 (14.9)	83 (16.9)	79 (13.5)	180 (11.7)	
Chemotherapy and hormonal therapy	2427 (31.0)	173 (35.2)	255 (43.5)	662 (43.1)	
Radiotherapy	7820 (100)	492 (100)	352 (60.1)	786 (51.2)	<0.001

*pT* pathological tumor stage, *pN* regional lymph nodes stage

^a^Re-excision by BCS in 268 (45.7%) patients of whom all had radiotherapy (100%) and re-excision by mastectomy in 318 (54.3%) patients of whom 84 (26.4%) had radiotherapy

^b^Re-excision by BCS in 516 (33.7%) patients whom all had radiotherapy (100%) and re-excision by mastectomy in 1016 (66.3%) patients of whom 270 (26.5%) had radiotherapy

^c^Includes mixed ductal and lobular tumors

^d^Both neoadjuvant and adjuvant

#### Ipsilateral breast tumor recurrence (IBTR)

After a median follow-up time of 60 months (IQR 59–60), 222 IBTR’s occurred. Five-year IBTR rate was 2.3% in patients with negative margin and primary BCS only, 2.9% in patients with focally positive margin and primary BCS only, 1.1% in patients with focally positive margin and re-excision, and 2.9% in patients with extensively positive margin and re-excision (*p* = 0.099) (Table 4). Interaction between all clinicopathological and treatment characteristics with margin status was tested but none interacted statistically significantly. Multivariable analyses showed that performing re-excision in patients with focally positive margins was statistically significantly associated with a lower IBTR rate as compared to omitting re-excision (adjusted HR 0.30 95% CI 0.11–0.82); however the absolute difference in 5-year IBTR rate was low 1.8% (2.9–1.1%).

**Table 4:** Ipsilateral breast tumor recurrence (IBTR), disease-free survival (DFS), and overall survival (OS) rates from Kaplan–Meier analysis and adjusted hazard ratio (HR) from multivariable Cox regression analysis according to resection margins after primary BCS and the performance of re-excision in the subcohort (*n* = 10433)

	IBTR		DFS		OS		
	5-year	Adjusted^a^ HR (95% CI)	5-year (%)	Adjusted^a^ HR (95% CI)	5-year (%)	10-year (%)	Adjusted^a^ HR (95% CI)
Negative margins and primary BCS only (*n* = 7820)^b^	163 (2.3%)	0.76 (0.43–1.35)	89.2	0.81 (0.63–1.04)	93.1	82.9%	1.07 (0.85–1.34)
Focally positive margins							
Primary BCS only (*n* = 492)^b^	13 (2.9%)	Reference	86.0	Reference	92.7	81.1	Reference
Primary BCS + re-excision (*n* = 586)^c^	6 (1.1%)	*0*.*30 (0*.*11*–*0*.*82)*	87.1	0.83 (0.59–1.17)	92.1	82.1	1.17 (0.87–1.59)
By BCS (*n* = 268)^b^	3 (1.3%)	0.39 (0.11–1.38)	90.7	0.66 (0.42–1.06)	94.8	86.0	1.01 (0.68–1.49)
By mastectomy (*n* = 318)^d^	3 (1.0%)	*0*.*23 (0*.*06*–*0*.*89)*	84.0	0.98 (0.66–1.47)	89.9	78.7	1.34 (0.93–1.91)
Extensively positive margins and primary BCS + re-excision (*n* = 1535)^e^	40 (2.9%)	0.75 (0.37–1.51)	84.7	0.97 (0.72–1.31)	91.8	81.1	1.22 (0.94–1.59)

Italic values indicate* p* < 0.05

^a^Adjusted for: age (continuous), histology (ductal, lobular, or other), differentiation grade (1, 2, 3, or unknown), pT stage (1, 2, 3, or ypT0), pN stage (1, 2, 3, or unknown), estrogen receptor status (positive, negative, or unknown), HER2Neu receptor status (positive, negative, or unknown), use of systemic therapy (any or none), and radiotherapy (yes or no). Complete table can be found in the Online Resource 1, Table 1

^b^Radiotherapy was performed in all patients with BCS (100%)

^c^Radiotherapy was performed in 352 (60.1%) of the patients since all 268 patients with re-excision by BCS (100%) and 84 (26.4%) of the patients with re-excision by mastectomy had radiotherapy

^d^Radiotherapy was performed in 84 (26.4%) patients

^e^Adjuvant radiotherapy was performed in 786 (51.2%) patients since all 516 patients with re-excision by BCS (100%) and 270 (26.5%) of the patients with re-excision by mastectomy had radiotherapy

#### Disease-free survival (DFS)

After a median follow-up time of 60 months (IQR 59–60), 1181 patients developed recurrent disease. Five-year DFS rates are shown in Table 4. Multivariable analyses showed that performing re-excision in patients with focally positive margins was not associated with improved DFS as compared to omitting re-excision (adjusted HR 0.83 95% CI 0.59–1.17).

#### Overall survival (OS)

After a median follow-up time of 110 months (IQR 78–135), 1709 deaths of any cause occurred. Five- and 10-year OS rates are shown in Table 4. Multivariable analyses showed that performing re-excision in patients with focally positive margins was not associated with improved OS as compared to omitting re-excision (adjusted HR 1.17 95% CI 0.87–1.59).

#### Systemic therapy

Stratifying the subcohort into patients who did (6398 patients) and did not (4035 patients) had (neo) adjuvant systemic therapy showed that 5-year IBTR rate was always lower than 4.0% independent of margin status and use of re-excision (see Online Resource 1, Table 2). In patients with systemic therapy, performing re-excision for focally positive margins was not statistically significantly associated with lower IBTR as compared to omitting re-excision (unadjusted HR 0.28 95% CI 0.08–1.06). In patients without systemic therapy, performing re-excision for focally positive margins was not statistically significantly associated with lower IBTR as compared to omitting re-excision (unadjusted HR 0.61 95% CI 0.15–2.56). After selecting patients with focally positive margins and primary BCS only, use of systemic therapy was not statistically significantly associated with lower IBTR compared to no use of systemic therapy (unadjusted HR 0.92 95% CI 0.30–2.81).

## Discussion

Omitting re-excision for focally positive margins was associated with statistically significantly higher IBTR rate as compared to performing re-excision (adjusted HR 0.30 95% CI 0.11–0.82), but the absolute difference was small (1.8% at 5-years), the absolute number of events was already low in both groups (2.9% versus 1.1% at 5-years), and the odds ratio was not significantly different from negative margins. Moreover, omitting re-excision in case of focally positive margins did not adversely affect DFS and OS. In the total study population (*n* = 32119) irrespective of the margins, IBTR rate was similar for patients with primary BCS only and patients with re-excision. Therefore, it can be concluded that omitting re-excision for focally positive margins does not impair DFS and OS. It could be argued that the difference found in IBTR rate is not clinically relevant; however, more evidence is needed to confirm this. Introducing the policy to omit re-excision for focally positive margins could potentially prevent large numbers of mastectomies which accounted for over 50% of the re-excisions. Interestingly, comparing IBTR, DFS, and OS rates between the re-excision by BCS group and re-excision by mastectomy group in the total study population (Table 2), it suggests that mastectomy is not preferred over BCS. Less mastectomies and attendant breast reconstructions will reduce burden to the patient and health-care costs that have been estimated to be $1055 per patient attempting BCS [17]. The same holds for the costly procedure of cavity shaving that has recently been introduced since our high rate of local control in patients with involved margins suggests it is unnecessary [18, 19].

Both the SSO/ASTRO guideline and the meta-analysis that was the guideline’s primary evidence base do not separate positive margins into focally or extensively positive. Six studies that did describe prognosis in patients with focally positive margins previously were all unicenter, predominantly from the 1980s, and margin status was defined *after* the final surgery and not after the *first* surgery [4–7, 9, 10]. They included only between 10 and 124 patients with focally positive margins who had whole-breast irradiation with a total dose range 55–65 Gy. Five- and eight-year local recurrence rates were reported by four studies and two studies, respectively, and ranged between 2 and 15% and 10–14%, respectively. Five out of six studies found that margin status after final excision was *not* statistically significantly associated with local recurrence after unadjusted analyses. These studies were too small to compare their findings to ours.

Our hypothesis is that radiotherapy boost reduces IBTR rates and nullifies the prognostic influence of focally positive margins. Jones et al. showed that a positive margin after BCS not followed by a re-excision was *not* a risk factor for local relapse in the boost versus no boost trial [12]. All patients with final BCS in our study population underwent adjuvant whole-breast radiotherapy, since no radiotherapy after BCS was an exclusion criterion. Unfortunately, what patients actually received and if boost was included was not registered by the Netherlands Cancer Registry. The Dutch guideline has strict recommendations, however, regarding radiotherapy (see methods), but whether they were strictly followed is unknown. To estimate the frequency and height of boost received in our study population, all 21 radiotherapy institutes in the Netherlands were contacted and radiation oncologists were questioned about their institute’s treatment policy in 2003–2008. Sixteen (76.2%) responded and all reported to have used a boost in patients with focally positive margins in whom re-excision was omitted with a median of 20 Gy (range 14–26 Gy). In patients with focally positive margins in whom re-excision was performed, one institute reported never to have used a boost after 2004, seven institutes always used boost, and eight institutes omitted boost in older patients and/or took into account grade and lymphovascular invasion. If boost was given, the median dose was 16 Gy (range 14–26 Gy). Radiotherapy boost is not without costs both financial and cosmetic and often additional hospital visits are needed. Increasing dose is associated with increasing incidence of fibrosis [20]. However, no evidence is available determining if boost or re-excision is the least harmful or preferred by the patient.

The safety of omitting re-excision in case of focally positive margins could also be explained by increasing systemic therapy use and effectiveness over time which significantly improved local control after breast-conserving therapy. The 5-year local recurrence rate decreased from 9.8% in 1988–1998 to 3.3% in 2006–2010 in early stage breast cancer patients ≤40 years [21]. Moreover, in patients with focally positive margins, Park et al. described a local recurrence risk of 7% with systemic therapy as compared to 18% without systemic therapy [5]. Other studies evaluating the effect of systemic therapy in patients with focally positive margins do not exist as far as we know. In our study, 5-year IBTR rates in patients with focally positive margins in whom re-excision was omitted were low and not statistically significantly different both in the presence and absence of systemic therapy (2.8 and 3.2%, respectively), but the confidence intervals were wide due to low number of events. Progress in breast cancer screening and treatment including use of modern radiotherapy techniques, more effective systemic therapy, accurate radiological tumor localization, inking of surgical specimens, and adequate pathological examination of resection margins may explain why omitting re-excision for focally positive margins appears to be safe nowadays.

Firm conclusions cannot be drawn, since limitations apply to our retrospective study. Registration of resection margins was not mandatory for the registration clerks of the Netherlands Cancer Registry and was only available in 32.5% of the total study population. The availability of resection margins was not associated to a time period, hospitals, pathologists, or IBTR. Moreover, patients in whom resection margins was registered were comparable as far as patient, tumor and treatment characteristics are concerned (data not shown) and the incidence of focally positive margins was equal to a study of all breast-conserving surgeries for invasive cancer in 2012–2013 in the Netherlands (10.3 and 11.0%) [22]. This confirms that the subcohort is a random selection from the total study population. The caregivers motives for omitting or performing re-excision were unknown, which could have led to confounding. However, patients who did and who did not underwent re-excision for focally positive margins were comparable as far as patient, tumor and treatment characteristics are concerned (Table 3) and adjustment for all these possible confounders was performed in the multivariable analyses. This makes selection bias less likely, although unknown residual confounding may be present. A survey evaluating surgeons’ preferences for margins and re-excision found that—even though the new SSO-ASTRO guideline advises *against* re-excision in case of negative margin—12% would re-excise for triple-negative tumor within 1 mm, 50% would re-excise when imaging and pathology are discordant, when tumor was within 1 mm of multiple margins, and when multiple foci of DCIS extended to within 1 mm of multiple margins [23]. Similar considerations will apply to the Dutch clinical practice. Another limitation was the limited number of events impairing to study the effect of omitting re-excision in clinically relevant subgroups. Furthermore, the orientation of the involved margins was unknown, but it can be assumed that re-excision was omitted for anterior and posterior margin involvement if the standard full thickness breast tissue excision was performed which previously has been shown to result in satisfactory local control [24]. Another important issue is the incidence of a first IBTR after 5 years of follow-up, especially in the estrogen-receptor-positive tumors, and the changes in prognostic factors for IBTR related to the time of follow-up [25] [26]. This emphasizes the importance of longer follow-up to estimate effect of re-excision accurately.

Since a randomized controlled trial of surgical margins has never been performed and is unlikely to be realizable, reliance on observational data is an acceptable approach. No studies have been performed comparing prognosis in patients in whom the focally positive margin is defined after the *first* surgery and thus comparing a group without re-excision to a group with re-excision after first surgery. Moreover, this is the first nationwide population-based analysis on a detailed cancer registry database including all hospitals, enabling adjustment for confounders. We describe a 10–100 times larger study population as compared to previous studies even in an era with lower incidence of positive margins, an important strength our study. It is the first cohort completely treated in the 21st century approaching current daily practice in which large improvements in breast cancer treatment and overall prognosis have been accomplished.

Omitting re-excision in invasive breast cancer patients with focally positive margins after BCS does not impair DFS and OS. Provided that adjuvant whole-breast irradiation is given including boost to the tumor bed, more evidence is needed to confirm that IBTR is not impaired either. Adoption of this recommendation will lead to less re-excisions and mastectomies which have considerable clinical implications reducing patient discomfort, health-care costs, and possibly improvement of cosmetic result.

## Electronic supplementary material

Below is the link to the electronic supplementary material.

Supplementary material 1 (DOC 70 kb)
