# Supplementary material for: Omitting re-excision for focally positive margins after breast-conserving surgery does not impair disease-free and overall survival
Source: Breast Cancer Res Treat. 2017 Apr 7;164(1):157–67. doi: 10.1007/s10549-017-4232-6 (PMC5487695; doi:10.1007/s10549-017-4232-6)
Supplement: Supplementary file 1 — Supplementary material 1 (DOC 70 kb) [file 10549_2017_4232_MOESM1_ESM.doc]

Omitting re-excision for focally positive margins after breast conserving surgery does not impair disease free and overall survival. Breast Cancer Research and Treatment. Elvira L. Vos, Sabine Siesling, Margreet H.A. Baaijens, Cornelis Verhoef, Agnes Jager, Adri C. Voogd, Linetta B. Koppert. Department of Surgery, Erasmus MC Cancer Institute, PO Box 2400, 3000 CA, Rotterdam, the Netherlands. Email: l.koppert@erasmusmc.nl

**Online resource 1**

| **Table 1** Ipsilateral breast tumor recurrence (IBTR), disease free survival (DFS), and overall survival (OS) rates from Kaplan-Meier analysis and adjusted hazard ratio (HR) from multivariable Cox regression analysis (n = 10,433) | | | |
| --- | --- | --- | --- |
|  | IBTR | DFS | OS |
|  | Adjusted^a^  HR (95%CI) | Adjusted^a^  HR (95%CI) | Adjusted^a^  HR (95%CI) |
| Resection margins and surgical treatment |  |  |  |
| - negative margins and primary BCS only | 0.76 (0.43-1.35) | 0.81 (0.63-1.04) | 1.07 (0.85-1.34) |
| - focally positive margins |  |  |  |
| - primary BCS only | reference | reference | reference |
| - primary BCS + re-excision | *0.30 (0.11-0.82)* | 0.83 (0.59-1.17) | 1.17 (0.87-1.59) |
| - extensively positive margins and primary  BCS + re-excision | 0.75 (0.37-1.51) | 0.98 (0.73-1.32) | 1.22 (0.94-1.59) |
| Age | *0.98 (0.97-1.00)* | 1.00 (0.99-1.01) | *1.05 (1.05-1.06)* |
| Histology |  |  |  |
| - ductal | reference | reference | reference |
| - lobular | 0.87 (0.54-1.39) | 1.17 (0.98-1.40) | 0.93 (0.80-1.08) |
| - other | 0.86 (0.54-1.36) | 0.97 (0.80-1.19) | 0.94 (0.80-1.11) |
| Differentiation grade |  |  |  |
| - 1 | reference | reference | reference |
| - 2 | 1.26 (0.84-1.91) | *1.59 (1.31-1.92)* | *1.38 (1.19-1.60)* |
| - 3 | *2.63 (1.67-4.15)* | *2.62 (2.13-3.22)* | *2.02 (1.71-2.37)* |
| - unknown | 1.45 (0.78-2.63) | *1.50 (1.14-1.97)* | *1.40 (1.14-1.74)* |
| pT |  |  |  |
| - T1 | reference | reference | reference |
| - T2 | 1.30 (0.95-1.77) | *1.60 (1.40-1.82)* | *1.56 (1.40-1.74)* |
| - T3 | 1.48 (0.45-4.91) | 1.49 (0.94-2.35) | *1.89 (1.30-2.75)* |
| - ypT0 | - | 1.49 (0.47-4.67) | 1.54 (0.57-4.15) |
| pN |  |  |  |
| - N0 | reference | reference | reference |
| - N1 | 1.23 (0.85-1.78) | *1.41 (1.20-1.65)* | *1.37 (1.21-1.57)* |
| - N2 | *2.32 (1.34-4.01)* | *3.16 (2.57-3.89)* | *2.61 (2.18-3.13)* |
| - N3 | *4.19 (2.15-8.17)* | *5.51 (4.31-7.06)* | *4.45 (3.55-5.58)* |
| - unknown | 1.11 (0.27-4.54) | 1.19 (0.62-2.32) | *1.98 (1.34-2.94)* |
| Estrogen receptor |  |  |  |
| - positive | reference | reference | reference |
| - negative | *2.32 (1.49-3.59)* | *1.63 (1.33-2.00)* | *1.53 (1.26-1.86)* |
| - unknown | 2.43 (0.85-6.95) | 1.48 (0.96-2.29) | 1.12 (0.79-1.60) |
| Her2Neu receptor |  |  |  |
| - negative | reference | reference | reference |
| - positive | 1.10 (0.71-1.72) | 0.85 (0.70-1.04) | *0.78 (0.64-0.94)* |
| - unknown | 0.60 (0.21-1.74) | 0.86 (0.55-1.34) | 1.04 (0.73-1.50) |
| Systemic therapy (yes vs no) | *0.30 (0.20-0.44)* | *0.48 (0.41-0.58)* | *0.81 (0.71-0.93)* |
| Radiotherapy (yes vs no) | 0.67 (0.38-1.27) | 0.97 (0.76-1.23) | 1.11 (0.90-1.36) |
| ^a^Adjusted for: age (continuous), histology (ductal, lobular, or other), differentiation grade (1, 2, 3, or unknown), pT stage (1, 2, 3, or ypT0), pN stage (1, 2, 3, or unknown), estrogen receptor status (positive, negative, or unknown), her2neu receptor status (positive, negative, or unknown), use of systemic therapy (any or none), and radiotherapy (yes or no). | | | |

| **Table 2** Ipsilateral breast tumor recurrence (IBTR) rate from Kaplan-Meier analysis and unadjusted hazard ratio (HR) from univariable Cox regression analysis stratified for systemic therapy (chemotherapy and/or hormonal therapy) and according to resection margins after primary BCS and the performance of re-excision in the subcohort (n = 10,433) | | | | | | | |
| --- | --- | --- | --- | --- | --- | --- | --- |
|  | No systemic therapy | | | Systemic therapy | | | |
|  | n | 5-year | HR (95%CI) | | n | 5-year | HR (95%CI) |
| Negative margins and primary BCS only | 3214 | 2.7% | 0.87 (0.35-2.14) | | 4606 | 2.0% | 0.70 (0.34-1.45) |
| Focally positive margins |  |  |  | |  |  |  |
| - primary BCS only | 183 | 3.2% | reference | | 309 | 2.8% | reference |
| - primary BCS + re-excision | 170 | 1.9% | 0.61 (0.15-2.56) | | 416 | 0.8% | 0.28 (0.08-1.06) |
| Extensively positive margins and primary BCS + re-excision | 468 | 4.0% | 1.34 (0.49-3.63) | | 1067 | 2.3% | 0.85 (0.38-1.91) |
